# The pandemic within a pandemic: mental health and wellbeing of racially Minoritised women experiencing domestic abuse during the COVID-19 pandemic in the UK

**DOI:** 10.1186/s12905-024-03502-4

**Published:** 2024-12-23

**Authors:** Ankita Mishra, Jilly Gibson-Miller, Chantelle Wood

**Affiliations:** 1 Department of Psychology, University of Sheffield, Sheffield, UK; 2 School of Education, University of Sheffield, Sheffield, UK

**Keywords:** Domestic abuse, Racially Minoritised women, Mental health, Wellbeing, COVID-19 pandemic, Lockdown

## Abstract

**Background:**

The prevalence of domestic abuse is greater in times of humanitarian crisis, and the COVID-19 pandemic has been no different. Considerable evidence indicates that domestic abuse disproportionately impacts the mental health and wellbeing of racially Minoritised women. The present study aimed to explore racially Minoritised women’s experiences of domestic abuse and mental health in the COVID-19 pandemic in the UK.

**Method:**

An online cross-sectional survey was used with racially Minoritised women (*n* = 1202) in the UK during the third national lockdown.

**Results:**

Results demonstrate complex interplay of psychosocial factors, such as the roles of autonomy, resilience, self-silencing, family functioning, and social support as predictors of mental health and wellbeing during the ‘shadow pandemic’.

**Conclusion:**

Implications such as incorporating culturally competent mental health support, exploring the complex and multiple underpinnings of mental health in racially Minoritised victim-survivors of domestic abuse for future pandemic preparedness and support provision are discussed.

## Background

United Nations Women describes violence against women and girls as a fundamental violation of human rights that has short and long term consequences on women’s physical, mental, sexual and reproductive health [89]. According to the World Health Organisation, 1 in 3 women across the globe experience physical or sexual violence in their lifetime, primarily by an intimate partner [33]. In the UK, domestic abuse is defined as: ‘Any incident or pattern of incidents of controlling, coercive or violent and threatening behaviour, violence or abuse between those aged 16 or over who are or have been intimate partners, family members or relatives who are ‘personally connected’, regardless of gender, sexuality, ethnicity, religion or socioeconomic status’ [24]. This includes but is not limited to psychological, physical, sexual, financial, and emotional forms of abuse, honour based violence and Female Genital Mutilation (FGM). Extensive research has shown that domestic abuse is associated with adverse physical and mental health consequences which impact negatively on women’s physical and psychological quality of life, including bruising, gastrointestinal issues, broken bones, depression, suicidality, anxiety, low self-esteem, post-traumatic stress disorder (PTSD), sleep disorders, and substance abuse among women of all backgrounds [8, 15, 16], see [23] for review).

The prevalence of domestic abuse is often greater in times of humanitarian crisis [60]. Research from past disease outbreaks, such as Ebola Virus Disease (EVD) and Middle East Respiratory Syndrome (MERS) has recognised the differential impact of pandemics on women [62]. Furthermore, pandemics have been linked to increased violence against women through factors that contribute to a survivors’ inability to temporarily escape the abusive partner including economic vulnerability, limited mobility on account of quarantine and isolation; limited access to legal systems and support services; diminished access to health services; and changing law enforcement operations (see [68] for review). Literature also shows that it is the levels of uncertainty that emerge due to pandemics that increase stress/anxiety levels [54]. During the recent COVID-19 pandemic, many countries including the United Kingdom, United States, Brazil, and Australia reported a surge in cases of domestic violence [73, 80], due to mandatory home isolation and forced proximity with cohabiting perpetrator(s), physical and social distancing, financial uncertainties, and anxieties caused by the coronavirus [25, 94]. Indeed, UN Women described violence against women during the COVID-19 pandemic as a ‘shadow pandemic’, bringing attention to this urgent public health issue (UN [90]).

The COVID-19 pandemic is also likely to differentially impact vulnerable populations, including ethnically and/or racially Minoritised women [11, 34]. Recent research has provided preliminary evidence of this differential impact on the mental health of some disadvantaged and marginalised groups [54, 76]. Evidence suggests that even outside of the pandemic, racially Minoritised women are disproportionately impacted by domestic abuse [36, 47, 67] compared to White women [14, 20, 51, 82, 83]. In the UK, the latest data from the Office for National Statistics [63] estimates that rates of domestic abuse among racially Minoritised communities together is greater than White communities, with rates highest for Mixed ethnicity women (9.4%) followed by Black (4.6%) and Asian (4.4%) women, compared to White women (7.7%). However, these statistics are skewed by underreporting of domestic abuse in Minoritised communities who face prohibitive structural barriers [9, 69, 70]. In the present study, we argue that Black and Minoritised women experience unique forms of oppression and also respond to abuse in different ways due to the simultaneously intersecting nature of their racialised and gender identities [2, 32, 35]. Thus, it is important to view the experiences of domestic abuse of racially Minoritised women in crisis contexts through an intersectionality lens [22].

## Domestic Abuse, Mental Health and protective factors in Minoritised women

In addition to the greater risk of domestic abuse, racially Minoritised women are susceptible to multiple systemic challenges and social stressors which render them at a greater risk for poor mental health and wellbeing [42, 47, 66]. The Minority Stress Model [59] argues that sexual minorities are exposed to a more hostile and stressful social environment due to the experiences of discrimination, prejudice, and stigma, which disproportionately impacts their mental health. Similarly, for racially Minoritised women survivors of domestic abuse, the interlocking experiences of abuse, systemic racial health inequities, and experience of prejudice and discrimination in their broader social environment is likely to have a greater impact on mental health [12, 40, 59, 91].

A wealth of research demonstrates the impact of domestic abuse on Minoritised women’s mental health including higher rates of depression, anxiety, reduced wellbeing and poor mental health compared to those who haven’t experienced abuse [26, 72] as well as compared to White women with experiences of abuse [1, 14, 51]. The multiple risk factors of social isolation experienced during lockdowns along with the escalating racial health disparities together have the potential to magnify the distressing mental health consequences for Minoritised women observed during the pandemic [27]. Again, taking into consideration the layers of interlocking risk factors and social challenges that Minoritised women experiencing domestic abuse are exposed to during lockdowns will help enormously in developing tailored interventions for improving mental health in such women.

Research has identified protective factors that might mitigate the high levels of distress experienced by many survivors of abuse. Studies have highlighted the role of resilience [41], social support, [17] and autonomy [10] as likely buffers during such adverse situations. Resilience, the process of adapting well and bouncing back from any adversity, is associated with better mental health and wellbeing [95]. Similarly, a number of studies have identified social support as a key protective factor in the context of domestic abuse, aiding better mental health and wellbeing in Minoritised survivors [21, 64]. A recent study by Catabay et al., [18] suggests that social support and resilience could act as salient buffers against poor mental health in Black and Minoritised women who had experienced violence. Greater autonomy and agency has also been linked to more positive health outcomes for Minoritised women experiencing abuse [84, 96]. These studies suggest that resilience, autonomy, and social support may safeguard to some degree the mental health and wellbeing of Minoritised women experiencing abuse during the COVID-19 pandemic.

At the interpersonal level, Kang [46] has highlighted the need to consider family environment-related factors, including but not limited to sociodemographic features, relationships between family members, resources and stability of the family, in studies of violence against adults in the family. A multitude of studies have further shown a significant link between level and style of family functioning and mental health [19, 93, 97]. While some research has found associations between poorer family functioning and negative mental health consequences in the context of domestic abuse and partner violence [3, 48], there is little research exploring the dynamics of family relationships of racially Minoritised women experiencing abuse, and the possible impact of family functioning on their mental health and wellbeing.

Another significant factor influencing women’s mental health in the context of domestic abuse is silencing the self, an overarching concept that describes how women, based on gender norms and societal structures, actively ‘silence certain thoughts, feelings and actions’ to nurture and maintain intimate relationships [45], p. 98). Jack [43] argues that while women’s motivation to engage in self-silencing behaviours stems from the need to avoid further conflicts in intimate partner relationships, it also increases their risk of depression. Silencing of women who experience partner violence is linked with a complex interaction of interpersonal, environmental and sociocultural factors [70]. Some studies have shown a significant association of self-silencing with women’s mental health and wellbeing in the context of domestic abuse [56, 85]. Research has also found associations between negative mental health effects and self-silencing in intimate relationships across different racial groups [6, 38]. Jack and Ali [44] have further highlighted the significance of the social context in impaired mental health of those who engage in self-silencing across diverse cultures. It is therefore important to explore the self-silencing of racially Minoritised women experiencing abuse and its association with their mental health and wellbeing.

## The Present Study

The intersection of marginalisation and discrimination has made racially Minoritised women more susceptible to domestic violence during the COVID-19 pandemic [49, 78, 79], which has the potential to be debilitating for their mental health and wellbeing [57, 74]. This is an understudied research area and requires urgent attention. The present study explores the mental health and wellbeing of racially Minoritised women experiencing domestic abuse during the third national lockdown in the UK. First, we predict that there will be a difference in the mental health (operationalised as anxiety and depression) severity, wellbeing, and resilience between those participants who report domestic abuse and those who do not. We further seek to explore the role of a range of potentially mitigating psychosocial factors, including resilience, autonomy, silencing of the self, family functioning, and social support, influencing their mental health and wellbeing. Our second prediction is that for those Minoritised women experiencing abuse, self-silencing will be strongly correlated with their mental health and wellbeing; resilience, social support and autonomy will be positively correlated with their mental health; and family functioning will be negatively correlated with their mental health and wellbeing. Third, we hypothesise that autonomy, self-silencing, resilience, family functioning, and access to social support will be significant predictors of the mental health (i.e. anxiety, depression) and wellbeing of participants experiencing abuse.

The current study has been pre-registered on Open Science Framework (OSF): https://osf.io/pcrw7/?view_only=4e88eb2df08a4edbafb7c420be9333ac. We have altered the pre-registered hypotheses and analyses slightly to aid clarity and parsimony. This paper is a subset of the wider study that explored domestic abuse, mental health, and help-seeking patterns in racially Minoritised women during the UK lockdown in 2021. This paper sheds light on the mental health and wellbeing patterns of racially Minoritised women, while the whole study elucidates the domestic abuse, mental health and wellbeing and help-seeking patterns and predictors of racially Minoritised women during the third lockdown of the COVID-19 pandemic in the UK. The domestic abuse and help-seeking patterns and predictors have been reported in a separate paper (*Authors, in press*).

## Method

### Design

An online survey using a cross-sectional cohort design was employed to collect data on socio-demographic variables, mental health and wellbeing, silencing the self, family functioning, autonomy, experiences of domestic abuse, resilience, and social support. Participants took part in the study during the third national lockdown of the UK between February-July, 2021.

## Participants

Participants were 1202 racially Minoritised women (*M*age = 31.38 years, *SD*age = 9.46 years, Age range = 18—71 years; two participants did not report their age) in intimate partner relationships (e.g., married, cohabiting, civil partnership) and residing in the UK. 246 participants (20.5%) were Black women, 568 participants (47.3%) were Asian women, 291 participants (24.2%) were Mixed ethnic women, 97 participants (8%) were women from other Minoritised communities (e.g., Arab). See Table 1 for sample demographics. The survey period was from February to July 2021. The lockdown mandated that everyone stay at home with closure of schools, nurseries and non-essential retail, hospitality and other services were closed. People were allowed to only leave homes if they wanted to shop for basic necessities or exercise once a day within one’s local area. Data collection ended when lockdown measures were lifted.

**Table 1:** Characteristics of participants in terms of ethnicity and/race

Baseline characteristic	Experienced abuse atleast once during the lockdown		Did not experience any abuse during the lockdown		Did not answer the questions about abuse		Full sample	
	*n*	*%*	*n*	*%*	*n*	*%*	*n*	*%*
**Ethnic group**								
Asian/Asian British: Indian	118	9.82	66	5.49	3	0.25	187	15.56
Asian/Asian British: Pakistani	63	5.24	29	2.41	1	0.08	93	7.74
Asian/Asian British: Chinese	83	6.91	42	3.49	1	0.08	126	10.48
Asian/Asian British: Bangladeshi	34	2.83	8	0.67	1	0.08	43	3.58
Other Asian background	73	6.07	45	3.74	1	0.08	119	9.90
**Asian women (Total)**	371	30.87	190	15.81	7	0.58	568	47.25
Black (Caribbean)	41	3.41	19	1.58			60	4.99
Black (African)	74	6.16	60	4.99	1	0.08	135	11.23
Black (British)	37	3.08	7	0.58	1	0.08	45	3.74
Other Black background	5	0.42			1	0.08	6	0.50
**Black women (Total)**	157	13.06	86	7.15	3	0.25	246	20.47
Mixed ethnic (White and Black Carribean)	64	5.32	20	1.66	1	0.08	85	7.07
Mixed ethnic (White and Black African)	23	1.91	11	0.92			34	2.83
Mixed ethnic (White and Asian)	70	5.82	33	2.75			103	8.57
Other Mixed background	53	4.41	15	1.25	1	0.08	69	5.74
**Mixed ethnic women (Total)**	210	17.47	79	6.57	2	0.17	291	24.21
Arab	17	1.41	5	0.42	1	0.08	23	1.91
Any other	47	3.91	26	2.16			73	6.07
**All other minoritised women (Total)**	64	5.32	31	2.58	1	0.08	96	7.99
Missing data			1	0.08			1	0.08

A purposive and snowball sampling strategy was used. Participants were recruited via Prolific (an online participant recruitment platform), through charities/organisations working with victim-survivors of domestic abuse, racially Minoritised women centres and community groups, a range of social media platforms (e.g., Facebook, Twitter, Reddit). Invitations were also sent to Prolific Academic, networks and contacts of the research team, volunteers’ databases in the University and other University groups (e.g., the BAME Staff Network, BME Students’ Committee, etc.). In addition to the aforementioned purposive sampling, we also used the snowball sampling strategy by requesting those who took part in the study to share the survey link with others they knew who might be interested in being part of the study. Participants recruited through Prolific Academic were paid at the recommended rate of £7.50/hour for completing the survey. All other participants were given the option to enter a prize draw to win 1 of 25 £20 and 1 of 30 £10 online shopping vouchers.

Power analysis via G*Power (version 3.1) was conducted for all relevant analyses and the one with the larger sample size was regression analysis which indicated that 782 participants would provide 0.80 power to detect a small effect size (*r* = 0.10) at alpha = 0.05. Our target sample size was therefore 2346 (782 in each of the following ethnic categories, Black, Asian, Mixed ethnicity, Other Minoritised communities). We had not achieved our target sample size when the lockdown measures were lifted in July 2021. We therefore made a pragmatic choice to combine data from all ethnic groups in our analyses, in order to maximise power.

### Procedure

After gaining ethics approval from the Departmental Research Ethics committee of the University, participants were invited to take part in an online survey on the topic of ‘their home lives and relationships during the pandemic’, which they accessed via a link on an online recruitment invitation on Qualtrics. There was a pre-screening question in the survey about ethnicity and the platform, Prolific, also pre-screened participants before facilitating us with the recruitment of women of color participants.

After reading an information sheet and signing the consent form, participants then completed the survey. Questions were presented to all participants in the same order, designed to minimise the triggering nature of the survey. Specifically, the most sensitive items (domestic abuse questionnaires: CBS-R and CAS-SF; see below) were placed in the middle of the survey. There was no time limit to complete the questionnaire. The participants were given the option to close the browser if they wished to withdraw from the study, or return to it at a later time if they wished to. After completion, participants viewed a debriefing sheet and were signposted to a list of support/advice resources (eg., contact details of BME specialist domestic abuse services/charities, counselling helplines, mental health resources). Participants were also asked if they would be willing to pass the survey link on to others they knew who might be interested in taking part in the research.

## Measures

Survey measures relevant to the present study include the following:

### Socio-Demographic Characteristics

Participants were asked to report the following socio-demographic characteristics: age; ethnicity/race; religious beliefs; SES (measured through income levels); employment status; education levels, relationship status and length of current relationship; household make-up.i.e. number of individuals and children in the household; country of residence in the UK.

### Mental Health and wellbeing

Depression was measured using the depression subscale of the Patient Health Questionnaire (PHQ-9) [50]. This scale consists of nine items (e.g., “Over the past two weeks, how often have you been bothered by the following problems? Little interest or pleasure in doing things”), scored 0 (Not at all) to 3 (Nearly everyday). The total score was calculated by taking the sum of scores of all the 9 items, giving a severity score ranging from 0 to 27 and the final score was calculated by taking an average of all the 9 items. Higher scores indicate increasing severity of depression. In line with Kroenke and Spitzer [50], total scores in the range of 0–4 are interpreted as no depression, 5–9 as ‘mild’, 10–14 as ‘moderate’, 15–19 as ‘moderately severe’ and 20–27 as ‘severe’ depression. Internal consistency in the current study was excellent (Cronbach α = 0.89) and similar to past research (Cronbach α ranging from 0.87 to 0.89: [50].

Generalised Anxiety Disorder-7 (GAD-7) [81] was used to measure symptoms of anxiety. This scale consists of seven items (e.g., “Over the past two weeks, how often have you been bothered by the following problems? Not being able to stop or control worrying”), scored 0 (Not at all) to 3 (Nearly everyday). The total score was calculated by taking the sum of scores of all the 7 items, giving a severity score ranging from 0 to 21 and the final score was calculated by taking an average of all the 7 items. Higher scores reflect increasing severity of anxiety. In line with Spitzer et al., [81], scores in the range of 0–5 have been interpreted as ‘mild’, 6–10 as ‘moderate’, 11–15 as ‘moderately severe’ and 16–21 as ‘severe’ anxiety. Internal consistency in the current study was excellent (Cronbach α = 0.92) and consistent with past research (Cronbach α = 0.92: [81].

WHO-5 Well-Being Index [7] is a 5 item questionnaire that was used to measure participants’ general wellbeing levels (e.g., “Over the past two weeks, how often have you experienced the following: I have felt calm and relaxed.”), scored on a 6 point Likert scale ranging from 0 (At no time) to 5 (All of the time). Higher scores reflect greater wellbeing and quality of life. We found excellent internal consistency of the scale (Cronbach α = 0.92) in our study.

### Silencing The Self

The Silencing The Self Scale [45] is a 31 item questionnaire which was used to measure normative beliefs in intimate-partner relationships that are considered “socially desirable” for women (e.g., “In a close relationship my responsibility is to make the other person happy”). Each item is scored on a 5-point scale ranging from 1 (Strongly disagree) to 5 (Strongly agree), with some items being reverse scored. Higher scores indicate greater pressure to fulfil the role of a “good woman” in the relationship. We found excellent internal consistency of the scale (Cronbach α = 0.91), similar to past research (Cronbach α ranging from 0.86 to 0.94: [45].

### Family Functioning

The level of family functioning was measured using the Brief Family Relationship Scale (BFRS) which is adapted from the 27-item Relationship dimension of the Family Environment Scale (FES) developed by [61], consisting of cohesion, expressiveness and conflict subscales. The BFRS is a 19 item scale (e.g., “In our family we really help and support each other a lot”; “In our family, we argue a lot”) which asked participants to respond how frequently such was the case in their family during the lockdown [29]. Each item is scored on a 3 point scale ranging from 0 (Not at all) to 2 (A lot), with some items being reverse scored. Higher scores indicated better family functioning. The calculated Cronbach α = 0.92 reflects excellent consistency for the scale in our study.

### Autonomy

In order to measure the degree of empowerment, agency and autonomy participants have in their own life, a template based on the definition and components of autonomy developed by Centre for Analysis of Social Exclusion, University of Oxford [13] was used in the present study. It was measured using 6 items from the template about autonomy in decision making, quality of options in life (e.g., “I feel like I am free to decide for myself how to live my life.”), with each item scored on a 5 point Likert scale ranging from 1 (Strongly Agree) to 5 (Strongly Disagree); 2 items about autonomy in relationships (e.g., “Do you feel free to form or maintain a relationship with someone of your choosing without external pressures?”) where each of the items is scored on a 5 point Likert scale ranging from 1 (Never or almost never) to 5 (Always or nearly always) and 1 item about the relevance of improving autonomy in relationships for the participants (e.g., “How important would it be for you to see an improvement in this aspect of your life?”) which was also scored on a 5 point rating scale ranging from 1 (Not important at all) to 5 (Very important). The final score was calculated by taking an average score on all the 9 items with higher scores reflecting greater choice and autonomy in the lives of the participants. In our study, the calculated Cronbach α = 0.75 indicates good internal consistency of the questions on autonomy.

### Domestic Abuse

Two domestic abuse screening instruments were used in our questionnaire, namely, the Composite Abuse Scale (Revised)-Short Form (CASR-SF) and the Controlling Behaviours Scale-Revised (CBS-R), to assess whether and to what extent the participants have experienced any form of abusive behaviours from their partner and/family member(s) during the lockdown.

The Composite Abuse Scale (Revised)—Short Form (CASR-SF) is a 15 item questionnaire measuring intimate partner violence by assessing physical (e.g., “My partner shook, pushed, grabbed or threw me”), sexual (e.g., “My partner made me perform sex acts that I did not want to perform”) and psychological abuse (e.g., “My partner blamed me for their violent behaviour”), with a focus on severity and intensity of experiences [30]. The participants were first asked if they had experienced each of the behaviours (Yes or No were scored as 1 or 0, respectively) during the pandemic. The total score on this question was calculated by taking a sum of the scores on all the 15 items. The total scores ranging from 0–15 were further coded into two categories, namely, No abuse at all (coded as 0) for obtained total scores of 0 and Presence of at least one abusive behaviour (coded as 1) for obtained scores ranging between 1–15.

Those who had responded ‘Yes’ to each of the abusive behaviours (participants’ with scores ranging from 1–15) were asked to rate how frequently they experienced those behaviours during the past 12 months, using the options: ‘not in the past 12 months’ (scored as 0), ‘once’ (scored as 1), ‘a few times’ (scored as 2), ‘monthly’ (scored as 3), ‘weekly’ (scored as 4), ‘daily or almost daily’ (scored as 5). The final frequency of abuse score was calculated by taking the average score of all the 15 items on this scale of 0–5. Higher scores indicated greater frequency of physical, sexual and psychological abuse experienced by the participants.

The Controlling Behaviours Scale-Revised (CBS-R), a 24 item questionnaire that measures controlling behaviours in the context of intimate-partner relationships across five subscales: Economic (e.g., “Refuse to share money/pay fair share”), Threats (e.g., “Threaten to disclose damaging or embarrassing information about you”), Intimidation (e.g., “Smash your property when annoyed/angry”), Emotional (e.g., “Tell you you were going mad”), and Isolation (e.g., “Try to limit the amount of activities outside the relationship”). Participants were asked to rate on a 5 point Likert scale how frequently they experienced those behaviours in the pandemic ranging from 0 (Never) to 4 (Very Often) [37]. The total scores ranged from 0–96 which were again coded into two categories, namely, No abuse at all (coded as 0) for obtained total scores of 0 and Presence of any of the abusive (controlling) behaviours at least once (coded as 1) for obtained scores ranging between 1–96. Higher scores indicated greater frequency of abuse experienced by the participants in the form of controlling behaviours by their partners.

Participants who had responded to either of the scales with scores between 1–15 for the CASR-SF and 1–96 for the CBS-R behaviour during the pandemic were categorised into ‘Presence of Abuse’ (coded as 1; *n* = 802) whilst those who had scored 0 on both the scales were categorised into ‘No Abuse at all’ (coded as 0).

### Resilience

We measured resilience using Brief Resilience Scale (BRS) [77] where participants responded to six items (e.g., “I tend to bounce back quickly after hard times”) on a five point Likert scale (Strongly Disagree—Strongly Agree). The total score ranged from 6–30. Higher scores on the scale indicated higher resilience among the participants. Our study found good internal consistency of the scale with Cronbach α = 0.86 similar to past research (Cronbach α ranging from 0.80–0.91: [77].

### Social Support

To measure access and availability of social support for the participants, we used the Inventory of Socially Supportive Behaviours-Short Form (ISSB-SF). The ISSB-SF is a 19-item self-report measure designed to assess ‘aid provision’ i.e. how often individuals received various forms of assistance such as directive guidance, tangible assistance, positive social exchange and the like in the past four weeks [5]. Participants were asked to rate how frequently other people did those activities (e.g., “Expressed interest and concern in your wellbeing”) for them on a 5 point Likert scale ranging from 0 (Not at all) to 4 (Almost everyday) and the final score was calculated by taking the average score of all the items, with higher scores indicating greater levels of availability of social support.

## Results

### Sample characteristics

Two-thirds of participants reported experiencing at least one abusive behaviour during the pandemic (66.7%; *n* = 802). Of these, 30.9% (*n* = 371) were Asian women, 13% (*n* = 157) were Black women, 17.5% (*n* = 210) were women of Mixed ethnicity and 5.3% (*n* = 64) were women from other Minoritised backgrounds. See Table 1 for further detail.

### Impact of Domestic Abuse on Mental Health and wellbeing

**Fig. 1:**
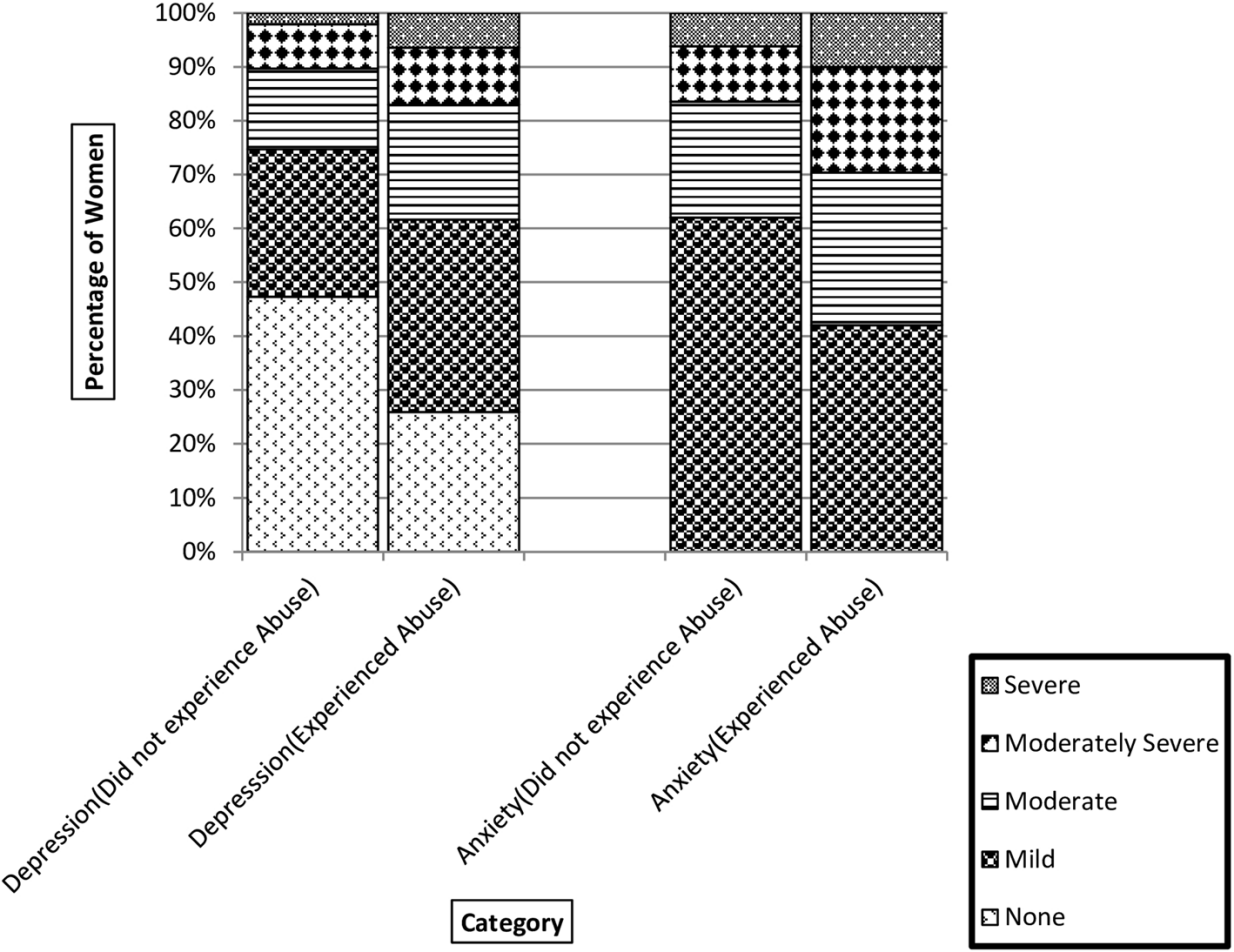
Graph representing mental health and wellbeing patterns of Minoritised women by the status of abuse during the lockdown

Scores on the PHQ-9, GAD-7, WHO-5 and BRS were each subjected to an independent t-test, with domestic violence experience (experienced some aspect of domestic abuse at least once during the pandemic vs. did not experience domestic abuse during the pandemic) as the independent factor. As shown in Table 2, participants who had experienced domestic abuse during the pandemic had significantly higher mean scores on the PHQ-9 and GAD-7, and significantly lower mean scores on the WHO-5 and BRS, relative to participants who had not experienced domestic abuse during the pandemic. Consistent with Hypothesis 1, results demonstrated significantly poorer mental health (depression and anxiety), wellbeing, and resilience amongst participants experiencing domestic abuse compared to those who did not. The total mean scores on PHQ-9, GAD-7 and WHO-5 are shown in Table 3, highlighting the difference in severity of mental health and wellbeing between participants experiencing domestic abuse compared to those who did not. Figure 1 compares percentages of women who reported experiencing abuse and did not experience abuse by severity category for depression (none, mild, moderate, moderately-severe, severe) and for anxiety (mild, moderate, moderately-severe, severe). The Figure clearly shows that there were greater proportions of women in the more severe categories for anxiety and depression for those women experiencing abuse compared to those who did not, suggesting that the experience of abuse led to more severe suffering. Although there was a greater proportion of all women reporting mild levels of anxiety (not an unexpected consequence in the context of the pandemic), the observed pattern for both anxiety and depression followed similar trends, whereby women experiencing abuse reached the threshold for more severe categories much sooner than women who did not report abuse. Nearly, one-third (29.6%) of women experiencing abuse, for example, reported being moderately or severely anxious compared to 16% of women not experiencing abuse. There was a similar difference (13% between groups) for combined moderate, moderate-severe and severe categories of depression.

**Table 2:** Results of t-test examining mental health, well being and resilience between participants who experienced abuse and those who did not

Variable	Those who experienced Abuse		Those who did not experience Abuse		t	P
	Mean	SD	Mean	SD		
Depression	0.98	0.66	0.7	0.61	6.955	< 0.001
Anxiety	1.09	0.78	0.73	0.75	7.647	< 0.001
Well Being	2.25	1.1	2.68	1.22	−5.868	< 0.001
Resilience	3.04	0.82	3.38	0.81	−6.716	< 0.001

**Table 3:** Summary of descriptive statistics for mental health of participants who experienced abuse and participants who did not experience abuse

Variable	Those who experienced Abuse		Those who did not experience Abuse	
	Mean	SD	Mean	SD
Depression	8.84	5.97	6.33	5.53
Anxiety	7.66	5.47	5.1	5.23
Well Being	11.24	5.51	13.39	6.09

### Influencing factors in mental health and wellbeing of Minoritised women experiencing abuse

For participants who reported experiencing domestic abuse during the pandemic, scores on the Silencing the Self scale, Autonomy scale, BRS, BFRS and ISSB-SF were subject to bivariate Pearson’s correlation with scores on the PHQ-9, GAD-7, and WHO-5. As shown in Table 4, scores on the Silencing the self scale were positively and moderately correlated with scores on the PHQ-9 and GAD-7, and negatively correlated with scores on the WHO-5; scores on the Autonomy scale, BRS and BFRS were negatively and moderately correlated with scores on the PHQ-9 and GAD-8, and positively correlated with WHO-5. Consistent with Hypothesis 2, therefore, the results suggested that for Minoritised women who experienced domestic abuse, increased silencing of the self, decreased autonomy, resilience, and family functioning was associated with increased depression and anxiety and decreased wellbeing. On the other hand, scores on ISSB-SF were positively and weakly correlated with scores on GAD-7 and WHO-5 and not correlated with scores on PHQ-9, indicating that increased access to social support was associated with increased anxiety and increased wellbeing and it did not have a significant relationship with depression.

**Table 4:** Descriptive Statistics and Correlations for Study Variables

Variable	***n***	***M***	***SD***	Depression	Anxiety	Well Being	Resilience	Self-Silencing	Autonomy	Family Functioning	Social Support
Depression	802	0.98	0.66	1	.78**	-.60**	-.41**	.43**	-.43**	-.28**	0.06
Anxiety	802	1.09	0.78	.78**	1	-.60**	-.42**	.40**	-.39**	-.25**	.14**
Well Being	802	2.25	1.10	-.60**	-.60**	1	.42**	-.33**	.35**	.22**	.09*
Resilience	801	3.04	0.82	-.40**	-.42**	.42**	1	-.29**	.33**	.18**	−0.06
Self-Silencing	802	2.71	0.57	.43**	.40**	-.33**	-.29**	1	-.51**	-.28**	-.09*
Autonomy	802	3.84	0.63	-.43**	-.39**	.35**	.33**	-.51**	1	.43**	.08*
Family Functioning	802	2.39	0.40	-.28**	-.25**	.22**	.18**	-.28**	.43**	1	.08*
Social Support	796	1.12	0.76	0.06	.14**	.09*	−0.06	-.09*	.08*	.08*	1

Note. ^*^p < .05. ^**^p < .01

For participants who reported experiencing domestic abuse at least once during the pandemic, multiple linear regression analyses were conducted, whereby, autonomy, silencing the self, resilience, family functioning, and access to social support were entered as predictor variables and mental health and wellbeing were entered as outcome variables. As can be seen in Tables 5 and 6, scores on Autonomy, Silencing the Self scale, BRS, BFRS and ISSB-SF significantly predicted scores on PHQ-9 and GAD-7 respectively, suggesting that poor mental health (i.e. increasing depression and anxiety) in the participants experiencing abuse was predicted by lower levels of autonomy, resilience, family functioning and greater self-silencing and increased access to social support. Table 7 shows that scores on Autonomy, Silencing the Self scale, BRS and ISSB-SF significantly predicted scores on WHO-5, while BFRS did not, suggesting that the wellbeing of the participants experiencing abuse was predicted by higher levels of autonomy, resilience, social support and lower silencing of the self, while family functioning did not play any role.

**Table 5:** Multiple Linear Regression: Variables predicting Depression in participants experiencing abuse

Variables	B	SE B	Beta
Autonomy	−0.189	0.039	−0.18***
Silencing of Self	0.082	0.012	0.243***
Resilience	−0.305	0.039	−0.25***
Family Functioning	−0.077	0.026	−0.099**
Social Support	0.037	0.012	0.089**

Note. R^2^ = 31.3%

^*^p < .05. ^**^p < .01, ^***^p < .001

**Table 6:** Multiple Linear Regression: Variables predicting Anxiety in participants experiencing abuse

Variables	B	SE B	Beta
Autonomy	−0.149	0.036	−0.154***
Silencing of Self	0.072	0.011	0.233***
Resilience	−0.305	0.036	−0.273***
Family Functioning	−0.06	0.024	−0.083**
Social Support	0.061	0.012	0.158***

**Table 7:** Multiple Linear Regression: Variables predicting Well Being in participants experiencing abuse

Variables	B	SE B	Beta
Autonomy	0.133	0.038	0.137***
Silencing of Self	−0.042	0.011	−0.136***
Resilience	0.365	0.038	0.324***
Family Functioning	0.045	0.025	0.062
Social Support	0.03	0.012	0.078**

Note. R^2^ = 24.6%

^*^p < .05. ^**^p < .01, ^***^p < .001

## Discussion

The aim of this study was to explore the mental health and wellbeing of racially Minoritised women experiencing domestic abuse in the context of the UK COVID-19 pandemic. Our findings demonstrate that women who reported experiencing abuse during the pandemic had significantly poorer mental health and wellbeing than those who did not experience any abuse. We also found that various factors at individual, interpersonal and social levels were associated with the mental health and wellbeing of those who reported experiencing abuse. While higher levels of resilience, autonomy and family functioning significantly predicted better mental health and wellbeing of those who experienced abuse; increased self-silencing and greater access to social support significantly predicted poorer mental health and wellbeing for those women.

The present study provides insight into the experiences of racially Minoritised women during the COVID-19 pandemic in the UK, where nearly 67% of the sample reported experiencing at least one abusive behaviour in their domestic spheres. The pattern, severity and extent of mental health, wellbeing, and resilience of Minoritised women reporting domestic abuse we observed was notable in comparison with women who did not report experiencing any abuse during the pandemic. Higher levels of depression and anxiety, and lower levels of wellbeing and resilience were found in those who experienced abuse vs those who did not. This broadly supports previous studies which, outside of the pandemic, reported poorer mental health and wellbeing among women of colour who experienced abuse as opposed to those who did not [26, 51, 72]. It is also in line with preliminary findings of some studies which have highlighted the critical impact of the COVID-19 pandemic on various disadvantaged groups [11, 76].

Our findings may be explained in light of the minority stress model [59], suggesting a dynamic interaction of multiple structural and social stressors with the experiences of domestic abuse being further compounded by the social isolation of the stay-at-home conditions imposed during the pandemic. The increased severity of poor mental health of women experiencing domestic abuse highlights the urgent need to account for mental health needs in the domestic abuse response strategy during crisis situations, such as a pandemic. Further, our findings have important implications for policy and practice necessitating the integration of culturally competent mental health support within both formal and informal support networks for Minoritised domestic abuse survivors.

Our findings demonstrate the protective roles of resilience and autonomy for racially Minoritised survivors of domestic abuse during the pandemic in predicting better mental health and wellbeing. This aligns with a wide range of evidence that has shown that higher levels of resilience predicts positive and better mental health and wellbeing in survivors of abuse [55, 75, 95], and higher levels of autonomy is associated with improved wellbeing, reduced trauma in a variety of contexts, and more positive outcomes for health [10, 84, 96]. The current study further demonstrates that higher levels of family functioning also predict better mental health of Minoritised domestic abuse survivors under lockdown. These results are consistent with a multitude of studies that have shown a significant link between the level of family functioning and mental health in other contexts [19, 97]. We suggest that all of these protective factors together need to be bolstered during conditions of quarantine and lockdown to mitigate the negative effects of domestic abuse on Minoritised women’s mental health and wellbeing in this altered social context. Recommendations for policy and practice include developing and improving resources, interventions and services that can strengthen resilience, autonomy, and family functioning, and are culturally tailored to address the specific mental health needs of Minoritised women.

Consistent with the literature on racially Minoritised women’s self-silencing and mental health outside of the pandemic [6, 56], the present study also found that those participants who expressed greater self-silencing in their intimate relationships reported poorer mental health and wellbeing. Jack and Ali [44] argue that the social context is most significant in the relationship between women’s mental health and their tendency to silence themselves and we believe that the observed pattern of results here may be explained by the exacerbation of stereotyped beliefs about the gender roles and expectations in close relationships in this unique social context [28]. This finding further raises intriguing questions regarding the individualised and decontextualised conception of mental health and wellbeing and underscores the critical role of wider social and contextual factors in determining mental health status. To develop an in-depth understanding of mental health and wellbeing of Minoritised survivors of domestic abuse, future research should consider how ‘individual’ factors are shaped by social and relational contexts. Implications for practice include developing alternative models of community-based mental health support services that are easily accessible and address the wider contextual and social factors that impact the mental health and wellbeing of racially Minoritised domestic abuse survivors. Considerations for the domestic abuse policy landscape suggests the need to rethink the individualised and pathologised understanding of mental health and wellbeing of Minoritised survivors by taking ecological systems thinking approach to the issue.

While a number of studies have shown that social support has been associated with better mental health and wellbeing for Minoritised survivors of abuse [21, 31, 55, 64, 65, 86], the findings of the current study were at odds with our hypotheses. We found that higher levels of social support were weakly associated with higher rates of depression and anxiety, and predicted poorer mental health among women experiencing abuse. Recent studies in the pandemic context have found similar relationships between social support and mental health in different populations. In a US-based study with young adults during the COVID-19 pandemic, Longest and Kang [53] demonstrate that accessing online forms of social support is positively related to poorer mental health. Another study with Chinese adults during the COVID-19 pandemic suggests that increasing social support can have reverse buffering effects by enhancing associations of stress and mental health [52]. One potential explanation for our findings is that the challenges of accessing the changing nature and form of social support in lockdown conditions with the added demands of concealing such efforts from the perpetrator(s), might have augmented the already deteriorating mental health of the participants. This finding has important policy and practice implications for developing and reinforcing systems (e.g., remote communication applications) and ways (e.g., code-word schemes such as ASK for ANI in a UK pharmacy) of support seeking during crisis that enhance the ease of accessing social support while mitigating the concerns of being ‘found out’ by the perpetrator.

Furthermore, social conflict, defined as the stress, tension, and discord experienced by survivors of abuse within their social support networks seems to be widespread [87]. A number of studies have identified that informal and formal social support networks of domestic abuse survivors, such as family, friends, professionals, religious leaders, communities, and institutions can be intrusive, engage in sexism, systemic racism, victim blaming, minimising the abuse, add conditions to their offers of help and the like (see [4] for a review [58, 88, 92],). All of this has the potential to be perceived as unhelpful and may instead lead to social conflict and have a negative impact on the mental health of survivors [39]. We therefore propose that in addition to the taxing experiences of accessing support during lockdown, it is also possible that social conflict could be a potential factor that diminished the expected protective role of social support on the mental health and wellbeing of Minoritised survivors. In view of this, future research might consider exploring the multi-faceted nature of social support in the mental health and wellbeing of Minoritised survivors. Recommendations for policy and practice include equipping both formal and informal support providers in incorporating anti-racist, trauma-and-violence-informed, and culturally sensitive approaches in their support provision.

The results of the present study demonstrate how the mental health and wellbeing of Minoritised survivors of domestic abuse is influenced by psychosocial factors at multiple levels. We suggest that crisis situations, like the pandemic, interact with intersectional identities in complex ways to influence the nature and patterns of mental health in women of colour. We call for future research to take more critical and community-based approaches to mental health and account for complexity and context rather than an approach focused on the individual. Implications for policy, legislation and practice include recognising the multiple underpinnings of mental health and focus on enhancing protective factors whilst also simultaneously implementing systemic and structural changes as a means for improving mental health and wellbeing of racially Minoritised women experiencing domestic abuse. We also recommend the use of participatory research methods such as co-design workshops, mutual learning methods and community engagement practices to collaboratively engage diverse stakeholders to design interventions and recommendations for policy and practice that are relevant to Minoritised survivors’ lived experiences.

Limitations.

Despite surveying a large, ethnically diverse and representative community sample of Minoritised women, the accessibility, reach and sampling of the present study may have been limited due to its language (English only) and mode of availability (online, rather than paper-based) [71]. Further efforts are needed to amplify the ‘voices’ of women with diverse linguistic and digital accessibility needs. Equally, future research might also explore other intersectional aspects of identities of domestic abuse survivors such as sexuality, disability and its impact on mental health and wellbeing. The present study provides a snapshot of the mental health and wellbeing of all Minoritised survivors. Future research should take qualitative approaches to capture the nuances and manifold complexities of the lived experiences of women-of-colour survivors through their situatedness in multiple relational and social contexts. Qualitative approaches in future studies will also help amplify the ‘voices’ of racially Minoritised women victim-survivors of domestic abuse. Additionally, as this was a cross-sectional survey using purposive sampling providing evidence on the state of affairs during a specific UK lockdown, it is problematic to confirm causality or suggest generalisations. However, we hope that our findings do provide a foundation for important avenues of exploration for future longitudinal research.

## Conclusion

This study builds on existing knowledge in the literature in relation to racially Minoritised women’s experiences of domestic abuse and mental health in a unique social context of the UK COVID-19 pandemic. The findings demonstrate the roles of autonomy, resilience, self-silencing, family functioning and social support as predictors of mental health and wellbeing during the ‘shadow pandemic’ in dynamic and complex ways. Results also demonstrate the potential for developing future interventions by working with the Minoritised survivors, taking into account the interaction of individual, social, and contextual factors in mental health. Future longitudinal research can build on this research and increase its reach to Minoritised women through availability in multiple languages, modes and platforms.

## Data Availability

The datasets used and/or analysed during the current study are available from the corresponding author on reasonable request.
